# Symbiosis: In search of a deeper understanding

**DOI:** 10.1371/journal.pbio.3002595

**Published:** 2024-04-12

**Authors:** Thomas A. Richards, Nancy A. Moran

**Affiliations:** 1 Department of Biology, University of Oxford, Oxford, United Kingdom; 2 Department of Integrative Biology, University of Texas at Austin, Austin, Texas, United States of America

## Abstract

This editorial discusses a new collection of articles exploring emerging themes in symbiosis research, as researchers exploit modern research tools and new models to unravel how symbiotic interactions function and evolve.

## Teaser

How do distinct species cofunction in symbiosis, despite conflicting interests? A new collection of articles explores emerging themes as researchers exploit modern research tools and new models to unravel how symbiotic interactions function and evolve.

Symbiosis is a puzzle: seemingly in defiance of the view that biological organisms are selfish entities, nature has provided numerous examples of distantly related species “working together.” These ecological interactions have been fundamental to the emergence of Earth’s biological systems, for example, driving how eukaryotic cells evolved [1], how plants colonized the terrestrial biome [2], and how coral reef ecosystems are built [3]. Yet scratch below the surface, and collaborations between species are never fully cooperative. Many interactions remain transient maximizations of ecological, metabolic, or protective benefit. Even in long-established endosymbioses, in which a microbial symbiont lives within cells of the host, selection drives each species to evolve in directions that are often to their partner’s detriment [4]. Consequences for symbionts can include entrapment, genetic appropriation, genome reduction, loss of autonomy, expulsion, and being digested. And, from the host side, symbiosis can lead to obligate dependence on partners that are erratically present or that are mutation ridden and ecologically fragile. Such dynamics might appear to make these interactions inherently unstable, yet they arise frequently and often persist across considerable periods of time and many generations.

This fundamental dichotomy has fired interest in the study of symbiosis. Furthermore, we now know that, despite these destabilizing forces, symbiosis has generated radical evolutionary outcomes that have shaped the tree of life. Biologists are interested in the forces that drive the origins of symbioses and their subsequent evolution, and in symbioses that generate different kinds of outcomes, ranging from diversification and ecological success to extinction. For example, symbiosis has driven how eukaryotic cells relocate respiratory pathways [5] and how photosynthesis is co-opted, time and time again [1].

The field of symbiosis research crosses many disciplines and has a long history. For many decades, symbiosis research (such as the work of Wallin, one of the early proponents of the symbiotic ancestry of mitochondria) operated largely from the natural history perspective [1,6]. These early experiments were few in number and often turned out to be wrong, although sometimes the underlying hypothesis was later proven to be correct [6]. As part of this collection, John Archibald discusses the history of endosymbiosis research and its legacy across modern experimental and genomic endeavors [6].

Focus on patterns, phylogenetic and taxonomic, has been an important theme. With the growing use of genomic technologies across the biosciences, the focus in the field has shifted to understanding the genomic changes that correlate with the evolution of symbiotic interactions. One such theme is how the genome composition of endosymbiotic bacteria evolves in response to obligate associations within host cells. In some cases, these bacteria have undergone radical changes that challenge our current understanding of how cells operate. McCutcheon and colleagues [7] delve into this subject, exploring the question, “how can such systems be viable with so few genes.”

In a related theme, Brockhurst and collaborators discuss the role of mutational decay and antagonistic pleiotropy as microbes shift from free-living forms to symbiotic forms that are strictly vertically transmitted and obligately dependent on hosts [8]. They also discuss how both selection and synthetic consortia experiments can be informative for understanding the forces that drive the early mutational changes underlying symbiotic interactions.

The impact of microbiome studies, and the importance of symbiotic interactions mediated though microbiome interactions—an approach also underpinned by genomic technologies—has crystalized as a core theme. This is a subject discussed by Margaret McFall-Ngai with reference to animal–microbe interactions within the animal immune systems [9].

DNA sequences have revealed that symbioses are everywhere and have certain genomic features suggestive of functional roles, but these studies fall short of telling us how host and symbiont initially form an association or how they evolve to function together at different levels of intimacy and interdependence. One purpose of this collection is to set the scene for a growing trend in the field of cross-disciplinary experimental science using omics, cell biology, biochemistry, and mathematical modelling to explore the functional basis of symbiotic interactions. Within these wider trends, Souza and co-authors explore how mathematical modelling can be used to frame and explore questions regarding symbiotic interactions [10], outlining, for example, how Fermi estimates can be used to explore why prokaryote–prokaryote endosymbioses are so rare.

Finally, we invited a series of authors to tell us how emerging model systems can be used to tackle themes within the wider subject of symbiosis. In return, we got 4 Perspectives on emerging model systems that can be used to experimentally dissect organismal interactions. These 4 articles cover how choanoflagellates, the protistan sisters of the animals, respond to a growing list of bacterial chemical signals and yet largely live free of physical association with bacteria beyond the time-limited interaction of consuming them by phagocytosis [11]; how the *Aiptasia* anemone can be used to study the cellular functions that shape evolutionary trade-offs relevant to coral symbiosis [12]; how the core biology of the fungus *Rhizopus* is shaped by bacterial and viral interactions (for example, by determining sexual reproduction and the toxin production that is fundamental to the pathogenic functions of these fungi) [13]; and how the protist *Paramecium bursaria*, the “swimming bag of algae,” is enabling experimental approaches for illuminating how the host cell can control the fate of its intracellular endosymbionts [14].

The mechanistic biology that underpins symbiotic outcomes is fascinating. It is also where this field’s most interesting future lies. How do complex multifaceted symbiotic interactions emerge? How is partner specificity enacted? How is stability maintained under strong evolutionary imperative towards exploitation and, therefore, interaction collapse? This collection demonstrates how the field is shifting, with a growing focus on biological mechanisms that underpin symbiotic interactions. This is a good thing. By combining mechanistic and genetic understanding with evolutionary analysis, we can gain a direct view of how symbioses emerge. Only when we have this view for a range of systems can we look for unifying themes, if they even exist.
